# The Role of Microglial CX3CR1 in Schizophrenia-Related Behaviors Induced by Social Isolation

**DOI:** 10.3389/fnint.2020.551676

**Published:** 2020-09-04

**Authors:** Hao Zhou, Jiesi Wang, Yu Zhang, Feng Shao, Weiwen Wang

**Affiliations:** ^1^Beijing Key Laboratory of Behavior and Mental Health, School of Psychological and Cognitive Sciences, Peking University, Beijing, China; ^2^Key Laboratory of Mental Health, Institute of Psychology, Chinese Academy of Sciences, Beijing, China; ^3^School of Nursing, Binzhou Medical University, Yantai, China

**Keywords:** CX3CR1, microglia, schizophrenia, social isolation, prepulse inhibition

## Abstract

According to the microglial hypothesis of schizophrenia, the hyperactivation of microglia and the release of proinflammatory cytokines lead to neuronal loss, which is highly related to the onset of schizophrenia. Recent studies have demonstrated that fractalkine (CX3CL1) and its receptor CX3CR1 modulate the function of microglia. Thus, the present study aimed to determine whether microglial CX3CR1 plays a role in schizophrenia-related behaviors. A classical animal model of schizophrenia, social isolation (from postnatal days 21–56), was used to induce schizophrenia-related behaviors in C57BL/6J and CX3CR1^−/−^ mice, and the expression of the microglial CX3CR1 protein was examined in several brain areas of the C57BL/6J mice by Western blot analysis. The results revealed that social isolation caused deficits in the prepulse inhibition (PPI) in the C57BL/6J mice but not in the CX3CR1^−/−^ mice and increased locomotor activity in both the C57BL/6J mice and the CX3CR1^−/−^ mice. Moreover, the CX3CR1 protein level was increased in the medial prefrontal cortex, nucleus accumbens, and hippocampus of the isolated C57BL/6J mice. These findings suggested that the function of microglia regulated by CX3CR1 might participate in schizophrenia-related behaviors.

## Introduction

Schizophrenia is a complex and severe mental illness that has been predicted to be the second leading factor contributing to the global burden of disease by 2030 together with depression and addiction (Gordon and Dzirasa, 2016). There are three main features of schizophrenia: positive symptoms, negative symptoms, and cognitive deficits. The positive symptoms consist of hallucination and delusion, and the negative symptoms consist of social avoidance and depression (Agnieszka, 2018). Also, cognitive deficits are one of the core features (Lepage et al., 2014). Although the underlying mechanism of schizophrenia remains unclear, evidence has shown that neuroinflammation and immunogenetics might participate in the development of this disease (Keller et al., 2012). In 2009, Monji proposed the microglia hypothesis of schizophrenia in which microglia play an essential role in the development of schizophrenia (Monji et al., 2009). As a core immune component in the central nervous system (CNS), microglia monitor synapses, detect harmful agents and remove dead cells by suppressing or promoting neuroinflammation (Mizuno, 2015). Usually, microglia have a beneficial healing effect towards the damage in the brain, by keeping the balance of suppressing or promoting neuroinflammation. However, when the damage lasts for a very long period under certain conditions, the production of too many cytokines and the long-term effects of inflammation modulated by microglia may be toxic (Schlegelmilch et al., 2011), which could increase the risk of mental illnesses such as schizophrenia (Monji et al., 2009). For example, Doorduin et al. (2009) found a significantly higher binding potential of ^11^C-(R)-PK11195 (a peripheral benzodiazepine receptor ligand that can be used for the imaging of microglia cell) in the hippocampus of patients with schizophrenia, which indicated microglia and neuroinflammation might play an important role in schizophrenic patients during psychosis. Moreover, studies have shown that most patients with schizophrenia suffer from an imbalance in their immune function. For example, it was reported that there was a cytokine imbalance in people with schizophrenia, as *in vivo* IL-1RA, soluble IL-2 receptor, and IL-6 were found to be increased (Potvin et al., 2008; Keller et al., 2012). Similarly, Goldsmith et al. (2016) found in a meta-analysis study that levels of IL-6, TNF-α, sIL-2R, and IL-1RA were significantly increased in acutely ill patients with schizophrenia, Boerrigter et al. (2017) found that the anti-inflammatory IL-2 mRNA was decreased and pro-inflammatory cytokines IL-6, TNF-α were increased in the peripheral blood of people with schizophrenia. Consistent results have been found in animal studies. Juckel et al. (2011) reported less branched microglia in the hippocampus and corpus striatum of the offspring induced by the injection of PolyI:C into pregnant rats, which indicated a phagocytic state of microglia in schizophrenia-like animal model. These results have revealed that microglia might participate in the development of schizophrenia.

Thus, researchers have started to focus on a critical target that modulates the function of microglia, the chemokine adhered to the surface of microglia (Ślusarczyk et al., 2015). Among all the chemokines, CX3CR1 and its ligand CX3CL1 directly modulate the function of microglia (Wolf et al., 2013). CX3CL1 localizes in the brain parenchyma to selected neurons, while CX3CR1 exists mostly on the surface of microglia in the CNS (Wolf et al., 2013). CX3CL1 mRNA was found to be highly expressed in the olfactory bulb, cerebral cortex, hippocampus, caudate-putamen, and nucleus accumbens (Nishiyori et al., 1998; Ragozzino, 2002). There are two forms of CX3CL1: the soluble form and the membrane-bound form. The membrane-bound form of CX3CL1 can recognize damaged cells, and the soluble form can attract microglia to damaged cells by binding to CX3CR1 expressed on microglia. Recently, several studies have examined the modulation of CX3CR1 on microglia in patients with schizophrenia. Ishizuka et al. (2017) found that the Ala55Thr mutant downregulates CX3CL1–CX3CR1 signaling, which might be the cause of schizophrenia and autism spectrum disorders. Bergon et al. (2015) also found in a meta-analysis that CX3CR1 expression is dysregulated in the blood and brain of patients with schizophrenia. However, Zhang et al. (2020) found that there was no difference in the CX3CR1 mRNA expression in the anterior cingulate cortex between patients with schizophrenia and controls. There was even increased expression of CX3CR1 mRNA in the anterior cingulate cortex in schizophrenic patients without suicide compared with those who died by suicide (Zhang et al., 2020). These controversial results suggest that further studies are needed to reveal how CX3CR1 and microglia participate in the development of schizophrenia.

CX3CR1^−/−^ mice are a good model to determine whether microglia participate in the development of immune-related diseases. There have been many studies using CX3CR1^−/−^ mice in the field of stress, depression, and other immune-related physical diseases. For example, Winkler et al. (2017) used CX3CR1^−/−^ mice on C57BL/6 background in a stress-related study of microglia and found these mice to be resistant to a typical model of stress, including significantly decreased immobility and increased time of struggling in forced swimming and tail suspension tests. Hellwig et al. (2016) found that CX3CR1 deficient mice showed resistance to depressive-like behaviors and changes in microglia morphology in a particular chronic despair model, compared to wild-type mice. Rimmerman et al. (2016) reported that wild-type mice displayed reduced sucrose preference, impaired novel object recognition memory, and reduced neurogenesis in a chronic unpredictable stress model, while CX3CR1^−/−^ mice were completely resistant to these effects. Schubert et al. (2018) used CX3CR1^−/−^ mice in a study of immunomodulatory mechanisms of post-traumatic stress disorder and found that these mice showed decreased anxiety-like behaviors. Additionally, Yu et al. (2018) used CX3CR1^−/−^ mice and found that the CX3CL1–CX3CR1 axis together with the NF-κB signaling pathway participates in the development of fructose-induced kidney injury. However, until now, there has been a lack of research work to observe schizophrenia-like behaviors, such as prepulse inhibition (PPI), in CX3CR1^−/−^ mice.

Many models, such as maternal interference, early life interference, and social isolation, have been used to observe the schizophrenia-like behaviors of rats (Juckel et al., 2011; Wang et al., 2015). PPI is a typical cognitive function, and impaired PPI function is recognized as one of the most important features of patients with schizophrenia. Thus, impaired PPI is a key characteristic of a good animal model of schizophrenia (Braff et al., 2008; Tapias-Espinosa et al., 2018). In 1993, Geyer et al. (1993) reported that isolation-reared animals showed significantly decreased PPI. Our previous studies also proved that social isolation can efficiently induce deficits in the PPI (Han et al., 2011a; Li et al., 2016, 2019; Sun et al., 2017) and reversal learning (Han et al., 2011b). Hence, social isolation is an appropriate strategy to induce schizophrenia-like behavior.

Thus, in this study, we aimed to test whether CX3CR1 plays a role in the social isolation-induced schizophrenia-like behaviors. First, we investigated the effects of social isolation on the PPI and locomotor activity of C57BL/6J mice, and then, the expression of CX3CR1 in the mPFC, NAc, and HIP was measured. Second, the CX3CR1^−/−^ mice on a C57BL/6J background were used to further demonstrate the impact of impaired modulation of CX3CR1 on microglia in this process. This study might provide further support for the microglial hypothesis of schizophrenia and reveal a new target for future studies.

## Methods

### Experiment 1

#### Animals

Twenty-four specific-pathogen-free (SPF) male C57BL/6J mice were obtained from Vital River Laboratory Animal Technology Company Limited (Beijing, China). All mice were housed at 22 ± 2°C and a humidity of 40–60% in standard cages under a 12 h light/dark cycle (lights on between 7:00 and 19:00); and the cage measured 295 mm long * 190 mm wide * 125 mm high. Water and food were available *ad libitum*. The use of animals and the experimental procedures followed the National Institutes of Health Guide for the Care and Use of Laboratory Animals and were approved by the Institutional Animal Care and Use Committee (IACUC) of Peking University.

#### Social Isolation

At PND 21, all the mice were divided into two different rearing conditions (social rearing, SR, vs. isolated rearing, IR). All the mice were divided randomly to prevent litter effects. There were 10 mice in the social-rearing (SR) group, while the other 14 mice were in the isolated-rearing (IR) group. For the SR group, there were four mice in one cage, while for the IR group, there was only one mouse per cage. The two groups were reared separately for 5 weeks (PND 21–56). During this period, the mice in the IR group could only interact with others in visual, auditory, and olfactory ways but had no physical interactions. After 5 weeks, there was a handling process for 3 days. During this period, each mouse was put on the hand of the experimenter for 5 min per day. Then, behavioral tests were performed.

#### Behavioral Tests

##### Open Field Test for Locomotor Activity in the Animals

At PND 60, an open field test was performed to test the spontaneous locomotion of all the mice. This test was performed in an open, uniformly illuminated square box of 40 cm × 40 cm. The mice were allowed to walk freely for 15 min in the arena. The total distance the mice traveled was recorded by a LabState laboratory animal behavioral analysis system (AniLab ver 4.3, AniLab Scientific Instruments Company Limited).

##### The Acoustic Startle Response Test and PPI Test

At PND 62, acoustic startle and PPI tests were undertaken with an SR-LAB startle response system apparatus (San Diego Instruments, San Diego, CA, USA). Five of the 10 chambers of the apparatus were used to perform the experiment. Inside each chamber, there is a transparent plexiglass cylinder (interior diameter: 3.5 inches; length: 8 inches) used to keep the mouse still. The size of the cylinder was appropriately adjusted to prevent excessive restraint of the mouse, which could cause further stress. The startle response was quantified by a piezoelectric transducer beneath the cylinder, and the information on each move was displayed on a computer. During the whole session, there was a background noise of 66 dB. After an acclimation period of 5 min, 30 trials were conducted for each mouse. There were three kinds of trials: (1) one 40 ms startle pulse with a 120 dB noise; (2) one 20 ms prepulse with 74 dB and one startle pulse with 120 dB; and (3) one 20 ms prepulse with 82 dB and one startle pulse with 120 dB. All three kinds of trials were presented ten times with random sequences, and the interval between every two trials was also random, ranging from 10 to 30 s with an average of 20 s. The cylinder was cleaned with 75% ethanol between each session. The PPI percent was calculated as follows: %PPI = (1 − startle amplitude in the prepulse trial/startle amplitude in the pulse alone trial) × 100%.

The behavioral tests were designed and performed by two investigators, and the one ran the tests were blind to the experimental conditions. At 24 h after all the behavioral tests, all 24 mice were decapitated without anesthesia. The mPFC, NAc, and HIP tissues were carefully dissected and frozen for Western blot analysis.

#### Western Blot

The tissues of six mice in the IR group and six mice in the SR group were selected randomly for Western blot. All the tissues were lysed in RIPA buffer containing protease inhibitor cocktail (Roche, USA). After denaturation at 100°C for 10 min in 5* loading buffer (Solarbio, Beijing), total protein (15 μg) was loaded and separated on a 12% gel (Bio-Rad, Hercules, CA, USA) and then transferred to polyvinylidene fluoride (PVDF) membranes (Millipore, Kankakee, IL, USA) using Mini Trans-Blot Electrophoretic Transfer Cell (Bio-Rad, Hercules, CA, USA). For both CX3CR1 and GAPDH, the membranes were blocked at room temperature for 1 h in 5% fat-free milk in TBST buffer (150 mM NaCl, 50 mM Tris-HCl, 0.5% Tween-20, pH = 7.6), after which the membranes were incubated at 4°C overnight with the primary antibody. The primary antibody used for CX3CR1 was rabbit polyclonal anti-CX3CR1 (ab8021, 1:1,500, Abcam), and the one for GAPDH was mouse monoclonal anti-GAPDH (ab8245, 1:2,000, Abcam), and they were both diluted in 5% fat-free milk in TBST buffer. When the incubation ended, the membranes were washed with TBST three times to remove the primary antibody, each of which lasted 15 min. Then, the membranes were incubated with the secondary antibody at room temperature for 1 h. The secondary antibody used were anti-rabbit antibody (1:5,000, Zhongshanjinqiao, China) and anti-mouse antibody (1:5,000, Zhongshanjinqiao, China), which were diluted in 5% fat-free milk in TBST buffer. After incubation, the membranes were again washed with TBST three times. Then, the bands were visualized by ECL Western Blotting Substrate (Solarbio, Beijing), and the images were processed and acquired using ChemiDoc™ XRS + System (Bio-Rad, Hercules, CA, USA). The intensity of the bands was quantified using ImageJ software, and GAPDH was used as a loading control.

### Experiment 2

#### Animals

A total of 12 male CX3CR1^GFP/GFP^ mice in the genetic background of C57BL/6J were obtained from Dr. Bo Peng (The University of Hong Kong, Hong Kong, China), which were firstly imported from The Jackson Laboratory. There were six mice in the SR group, while the other six mice were in the IR group. All the mice were divided randomly to prevent litter effects. The rearing conditions were the same as those described in Experiment 1.

#### Social Isolation

At PND 21, the CX3CR1^−/−^ mice were divided into two groups and received the same rearing conditions as in Experiment 1.

#### Behavioral Tests

The two groups of CX3CR1^−/−^ mice both underwent the two behavioral tests as described in Experiment 1.

### Statistical Analysis

All analyses were performed using GraphPad Prism6 software (USA), and data are expressed as the mean ± standard error of the mean. The student’s *t*-test was used in the open field test and Western blots. Two-way ANOVA was used in the PPI test. The data which were three-fold standard deviation (SD) above or below the average were regarded as outliers and were dealt with. What’s more, two data from the 74 and 82 dB conditions of one control mouse were about 2.8-fold SD below the average in the PPI test. Considering the open field data of the same animal were also outliers, we dealt with these two data as outliers. The significance level was defined as *p* < 0.05.

## Results

### Social Isolation-Induced Increased Locomotor Activity in the Wild-Type Mice

**Figure 1:**
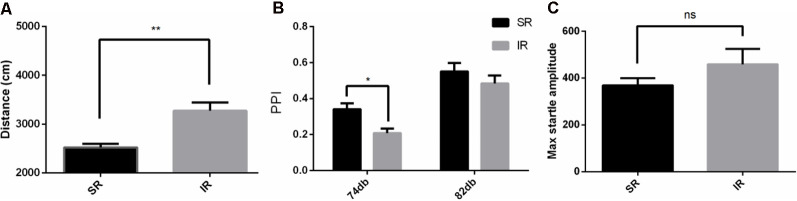
Effects of isolation rearing on the behaviors of the wild-type mice, including the total distance traveled **(A)** in the open field test, the prepulse inhibition (PPI)% **(B)** in the acoustic startle response and the PPI test, and the pulse-alone amplitudes, **(C)** in the acoustic startle response and the PPI test. The results are expressed as the mean ± SEM [*n* = 10 for the social-rearing (SR) group and *n* = 14 for the isolated-rearing (IR) group, *p < 0.05, **p < 0.01, ^ns^*p* > 0.05, student’s *t*-test and two-way ANOVA].

As shown in Figure 1A, in the open field test, the total distance traveled by the IR group was significantly longer than that traveled by the SR group (*t*_(19)_ = 3.331, *p* = 0.0035; If the outliers were included in the analysis, *t*_(22)_ = 1.815, *p* = 0.0831). Social isolation caused significantly increased spontaneous locomotion in the wild-type mice.

### Social Isolation Resulted in Impairments in the PPI in the Wild-Type Mice

As shown in Figure 1B, there was a significant effect of rearing condition between the SR group and IR group (*F*_(1,22)_ = 4.932, *p* = 0.0370; If the outliers were included in the analysis, *F*_(1,22)_ = 0.6050, *p* = 0.4450) and a significant effect of the startle condition between 74 and 82 dB (*F*_(1,22)_ = 66.06, *p* = 0.0001; If the outliers were included in the analysis, *F*_(1,22)_ = 59.97, *p* = 0.0001). There was no significant interaction between the rearing condition and the startle condition (*F*_(1,22)_ = 1.215, *p* = 0.2822; If the outliers were included in the analysis, *F*_(1,22)_ = 0.4099, *p* = 0.5287). As shown in Figure 1C, there was no difference in the pulse-alone trials between the SR group and IR group (*t*_(22)_ = 1.071, *p* = 0.2958). This result showed that social isolation impaired PPI.

### Social Isolation Increased the CX3CR1 Expression in the mPFC, NAc, and HIP

**Figure 2:**
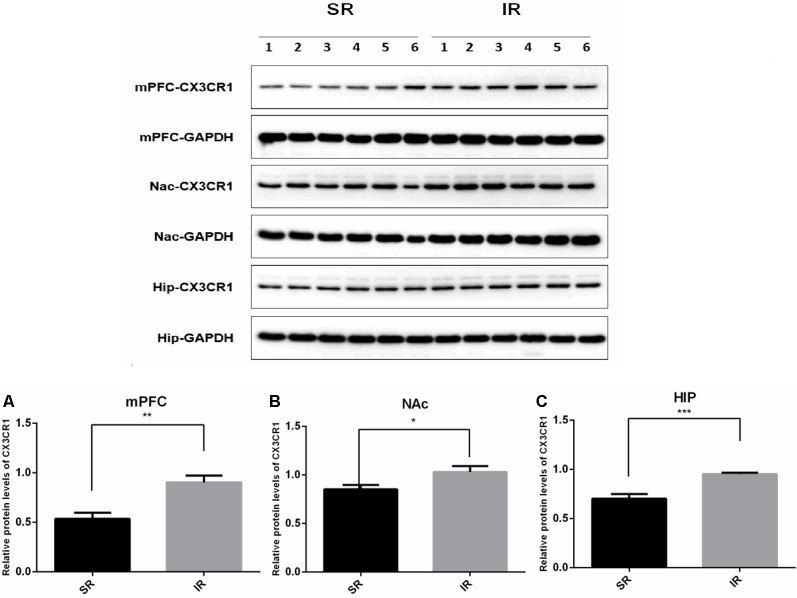
Effects of isolation rearing on the CX3CR1 expression in the wild-type mice. Different regions were tested, including the mPFC **(A)**, NAc **(B)**, and HIP **(C)**. The results are expressed as the mean ± SEM (*n* = 6 for the SR group and *n* = 6 for the IR group. *p < 0.05, **p < 0.01, ***p < 0.001, student’s *t*-test).

Western blot analysis revealed a specific band (≈50 kDa) of CX3CR1. As shown in Figure 2, there was a significantly higher expression of CX3CR1 in the mPFC **(A)** of the IR group than in the SR group (*t*_(10)_ = 3.932, *p* < 0.01), and there was a similar trend in the NAc **(B)** (*t*_(10)_ = 2.347, *p* = 0.0409) and HIP **(C)** (*t*_(10)_ = 4.862, *p* < 0.01).

### Social Isolation-Induced Increased Locomotor Activity in the CX3CR1^−/−^ Mice

**Figure 3:**
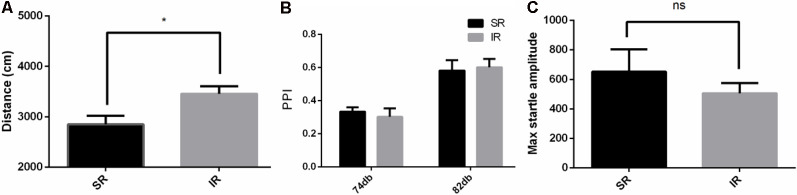
Effects of isolation rearing on the behavior of the CX3CR1^−/−^ mice, including the total distance traveled **(A)** in the open field test, the PPI%, **(B)** in the acoustic startle response and the PPI test, and the pulse-alone amplitudes, **(C)** in the acoustic startle response and the PPI test. The results are expressed as the mean ± SEM (*n* = 6 for the SR group and *n* = 6 for the IR group, *p < 0.05, ^ns^*p* > 0.05, student’s *t*-test and two-way ANOVA).

As shown in Figure 3A, in the open field test, the IR group of CX3CR1^−/−^ mice also traveled longer than the SR group (*t*_(9)_ = 2.594, *p* = 0.0290). Social isolation could induce increased spontaneous locomotion in the CX3CR1^−/−^ mice.

### Social Isolation Did Not Affect the PPI of the CX3CR1^−/−^ Mice

As shown in Figure 3B, there was a significant difference between the startle conditions (*F*_(1,9)_ = 99.71, *p* < 0.01), but there was no significant difference between the different rearing conditions of the CX3CR1^−/−^ mice (*F*_(1,9)_ = 0.008469, *p* = 0.9287), and there was no significant interaction effect (*F*_(1,9)_ = 0.8609, *p* = 0.3777). As shown in Figure 3C, there was no difference in the pulse-alone trials between the SR group and IR group (*t*_(9)_ = 0.8122, *p* = 0.4376). Social isolation did not cause significant deficits in the PPI of the CX3CR1^−/−^ mice compared to the wild-type mice.

## Discussion

The current study found that social isolation can induce schizophrenia-like behaviors in mice, similar to the results in rats. After isolated rearing, the C57BL/6J mice showed impaired PPI function and increased locomotor activity, similar to rats. Also, increased CX3CR1 protein levels in the mPFC, NAC, and HIP of the isolated C57BL/6J mice were observed. For the CX3CR1^−/−^ mice, social isolation-induced similarly increased locomotor activity but did not affect the PPI.

In our previous studies, isolation rearing was reported to induce increased locomotor activity and disrupted PPI function in rats (Han et al., 2011a; Li et al., 2016, 2019; Sun et al., 2017). Similar results were shown in experiment 1, where isolated rearing of the wild-type mice resulted in behaviors similar to those in rats. These results are consistent with previous studies using the model of social isolation of rodents (Abramov et al., 2004; Fone and Porkess, 2008; Huang et al., 2017). These findings proved that social isolation is a stable way to induce schizophrenia-like behavior in both rats and mice.

To investigate the effects of CX3CR1 on the schizophrenia-like behaviors induced by social isolation, we assessed the CX3CR1 protein levels within several regions, including the mPFC, NAc, and HIP, which are well-established sites for schizophrenia-like behavior. The results showed that the CX3CR1 protein was upregulated in all three areas of the wild-type mice. To the best of our knowledge, this is the first study to determine the effects of CX3CR1 in an animal model of PPI-related schizophrenia-like behaviors, and we could not compare the results with related animal studies. However, our results seem to be inconsistent with those of two other clinical studies. Bergon et al. (2015) found abnormal CX3CR1 mRNA expression in the blood and brain of patients with schizophrenia, while Zhang et al. (2020) reported that there was no significant difference between patients with schizophrenia and controls concerning the mRNA level of CX3CR1 in the anterior cingulate cortex. The most likely reasons for these complex and inconsistent results could be as follows. First, the evaluation might occur at a different stage of schizophrenia. As shown in Wynne’s study, there indeed was a dynamic change in CX3CR1 surface expression after activation. CX3CR1 expression may decrease first as a response to a stimulus, such as the injection of lipopolysaccharide. However, the reduction disappeared, and CX3CR1 expression returned to the former level in 24 h. Later, this reduction was even reversed, and CX3CR1 expression was found to be significantly increased after 24 h (Wynne et al., 2010). This finding suggested that the expression of CX3CR1 may differ in different stages of schizophrenia. Second, the expression of chemokines might not only be affected by transcriptional regulation but also translational regulation and translocation (Wynne et al., 2010). This phenomenon may explain the opposite results of the CX3CR1 mRNA levels and CX3CR1 expression (Ishizuka et al., 2017). In further studies, we will simultaneously test CX3CR1 mRNA, CX3CR1 protein, and the functional change of microglia to explore the covariation of these three factors. It has been reported that TGFβ might be a key factor that elevates the expression of CX3CR1 after its immediate downregulation and thus could be a new target in the future (Chen et al., 2003; Wynne et al., 2010). Determining the exact changes in both CX3CR1 and microglia may help us better understand the modulation of CX3CR1 on microglia and dynamic neuron-glia crosstalk.

By using CX3CR1^−/−^ mice, we found a similar influence of social isolation on these knock-out mice, where they presented similar increased locomotor activity. However, in contrast to the results in the wild-type mice, there was no significant impairment of the PPI function in the IR group compared to the SR group. Since it was revealed that the basic function of the acoustic response was not affected in CX3CR1^−/−^ mice (Schubert et al., 2018), this difference in isolation rearing-induced PPI deficit between the wild-type and CX3CR1^−/−^ mice could not be a result of the basic acoustic response deficit. The CX3CR1^−/−^ mice were reported to suffer from transiently reduced microglial densities in their first two postnatal weeks (Paolicelli et al., 2011), and this lack of microglia during early age caused microglial dysfunction, which might be related to the resistance to social isolation-induced impairments in the PPI.

Moreover, our results found that the locomotor activity was found to be increased in both genotypes while the PPI deficit was found to be CX3CR1-dependent. It suggested that the schizophrenia-like cognitive deficits such as PPI deficit seemed to be directly related to the function of microglia. This was similar to Rimmerman et al.’s (2016) conclusion that emotional and cognitive stress resilience might be a result of CX3CR1-dependent basal alterations in hippocampal transcription, for the result that the exposure of chronic unexpected stress could alter neuronal gene transcripts (e.g., Arc, Npas4) in both wild-type and CX3CR1^−/−^ mice, while the transcripts downstream of hippocampal estrogen receptor signaling were altered only in CX3CR1^−/−^ mice. While Ieraci et al. (2016) found in a chronic social isolation model of mice that the hyperactivity was correlated with the cortical BDNF-7 levels and metabotropic glutamate receptor mGluR1 in the PFC, Eßlinger et al. (2016) found that PPI deficits were preceded by a strong M1-type microglia polarization pattern in the whole brain during puberty in the model of prenatal Poly(I:C) injection. This might explain the difference between the CX3CR1-independent hyperactivity and CX3CR1-dependent PPI deficit. Further studies are required on the particular signaling pathway related to PPI deficit, CX3CR1, and microglia. Our study first used an animal model of schizophrenia to demonstrate that CX3CR1 might be a necessary factor in the development of schizophrenia-like behaviors, and the blockade of CX3CR1 might be useful in treating schizophrenia.

In conclusion, the present study found that social isolation can induce increased locomotor activity both in the C57BL/6J mice and the CX3CR1^−/−^ mice, and impairments in the PPI in the C57BL/6J mice but not in the CX3CR1^−/−^ mice. Moreover, CX3CR1 was upregulated in the mPFC, NAc, and HIP of the isolated C57BL/6J mice. These findings first demonstrated that CX3CR1 plays a role in the schizophrenia-like behaviors induced by social isolation, providing new directions for studies of microglial function in the development of schizophrenia and treatment strategies for schizophrenia.

## Data Availability Statement

The raw data supporting the conclusions of this article will be made available by the authors, without undue reservation.

## Ethics Statement

The animal study was reviewed and approved by Institutional Animal Care and Use Committee (IACUC) of Peking University.

## Author Contributions

FS and WW designed the study. HZ, JW, and YZ performed the research and obtained the data. HZ and JW interpreted and analyzed the data. HZ, WW, and FS drafted, revised, and wrote the article. All authors contributed to the article and approved the submitted version.

## Conflict of Interest

The authors declare that the research was conducted in the absence of any commercial or financial relationships that could be construed as a potential conflict of interest.
